# PDGFRβ-specific affibody-directed delivery of a photosensitizer, IR700, is efficient for vascular-targeted photodynamic therapy of colorectal cancer

**DOI:** 10.1080/10717544.2017.1407011

**Published:** 2017-11-28

**Authors:** Qiuxiao Shi, Ze Tao, Hao Yang, Qing Fan, Danfeng Wei, Lin Wan, Xiaofeng Lu

**Affiliations:** a Key Lab of Transplant Engineering and Immunology, MOH, West China Hospital, Sichuan University, Chengdu, China;; b Regenerative Medical Research Center, West China Hospital, Sichuan University, Chengdu, China;; c Medical Research Center, the Third People's Hospital of Chengdu, The Second Affiliated Chengdu Clinical College of Chongqing Medical University, Chengdu, China

**Keywords:** Photodynamic therapy, affibody, PDGFRβ, pericyte, cancer therapy

## Abstract

Vascular-targeted photodynamic therapy (PDT) is an important strategy for cancer therapy. Conventional vascular-targeted PDT has been achieved by passive photosensitizer (PS) delivery, which involves a high risk of adverse effects. Active PS delivery is urgently required for vascular-targeted PDT. Although endothelial cells and pericytes are major cellular components of tumor blood vessels, little attention has been paid to pericyte-targeted PDT for cancer therapy. PDGFRβ is abundantly expressed in the pericytes of various tumors. In this experiment, a dimeric Z_PDGFRβ_ affibody with a 0.9 nM affinity for PDGFRβ was produced. The Z_PDGFRβ_ affibody showed PDGFRβ-dependent pericyte binding. Intravenously injected Z_PDGFRβ_ affibody was predominantly distributed on pericytes and thus accumulated in LS174T tumor grafts. The conjugate of the Z_PDGFRβ_ affibody and IR700 dye, i.e. Z_IR700_, bound to PDGFRβ^+^ pericytes but not to PDGFRβ^−^ LS174T tumor cells. Accordingly, Z_IR700_-mediated PDT *in vitro* induced the death of pericytes but not of LS174T tumor cells. In mice bearing LS174T tumor grafts, Z_IR700_-mediated PDT damaged tumor blood vessels, thus inducing tumor destruction by intensifying tissue hypoxia. The average mass of tumor grafts administered with Z_IR700_-mediated PDT was approximately 20–30% of that of the control, indicating that pericyte-targeted PDT is efficient for cancer therapy. In addition, Z_IR700_-mediated PDT increased the tumor uptake of TNF-related apoptosis-inducing ligand (TRAIL) injected post-illumination. Consequently, combination therapy of Z_IR700_-mediated PDT and TRAIL showed greater tumor suppression than Z_IR700_-mediated PDT- or TRAIL-based monotherapy. These results demonstrated that active vascular-targeted PDT could be achieved by using Z_PDGFRβ_ affibody-directed delivery of PS.

## Introduction

Photodynamic therapy (PDT) is a two-stage procedure involving the administration of a photosensitizer (PS) followed by exposure to light. In the presence of tissue oxygen, the PS, activated by light of a specific wavelength, could stimulate the production of reactive oxygen species (ROS), thus inducing cell death (Dolmans et al., 2003; Dabrowski & Arnaut, 2015). Compared to conventional surgical interventions, PDT is minimally invasive for patients. In addition, PS, without excitation by light, is usually non-toxic. Moreover, due to the very short lifespan (<40 ns in biological systems) and a limited radius (<20 nm) of action of ROS, PDT-mediated photodamage can be restricted in disease sites by topical illumination. Unlike conventional chemotherapy, PDT involves a low risk of systematic toxicity and has thus been considered an ideal modality for the clinical treatment of cancers of the skin, brain, head, and neck, lung, esophagus, pancreas, bile duct, breast, bladder, prostate, stomach, colon, female reproductive tract, etc. (Shafirstein et al., 2017; van Straten et al., 2017).

In the case of passive PS-delivery-based PDT, although topical illumination reduced the risk of systematic toxicity, photosensitive reactions were usually observed in normal tissues, such as the skin and eyes, that are exposed to daylight (Bugaj, 2011). These side effects were attributed to the unspecific accumulation of free PS in these tissues. Consequently, tumor-targeted delivery of PS is required for PDT in the future (Bugaj, 2011; Shirasu et al., 2013). To achieve tumor-targeted delivery, passive and active targeting strategies have been developed in recent years. Passive tumor targeting might be achieved by exploiting the enhanced permeability and retention (EPR) effect caused by the leaky vasculature and impaired lymphatic drainage of tumors. In fact, most nanoparticles loaded with PS can be selectively accumulated in tumors due to the EPR effect (Lowa & Lin, 2000). Active tumor targeting was developed by the conjugation of PS to molecules that specifically bind antigens or receptors that are overexpressed in tumors. Thus far, antibodies and their fragments, peptides and protein ligands, and small molecules have been widely used for tumor-targeted delivery of PS (Schmitt & Juillerat-Jeanneret, 2012). Since passive targeting relies on only the EPR effect of tumors, its specificity and efficacy are limited. In contrast, active targeting depends on both the EPR effect and molecule–molecule interactions, which lead to more specific and effective delivery of PS to tumors (Shirasu et al., 2013).

Tumor cell targeting and vascular cell targeting are important strategies for active tumor targeting. Compared to tumor cell-targeted PDT, vascular-targeted PDT takes advantage of restricting nutrients and the oxygen supply, thus leading to stronger tumor growth suppression (Krzykawska-Serda et al., 2014), suggesting that vascular-targeted PDT is more efficient for cancer therapy. It is known that the walls of tumor blood vessels predominantly consist of irregularly lined endothelial cells and pericytes covering the endothelial tubule (Chang et al., 2013; Geevarghese & Herman, 2014). In previous works, vascular-targeted PDT has been achieved by the conjugation of PS to molecules that specifically bind vascular endothelial growth factor receptor (VEGFR) or αvβ3 integrin, which are overexpressed in tumor-associated endothelial cells (Thomas et al., 2010; Srivatsan et al., 2011). However, it is not known whether tumor-associated pericytes could also be considered target cells for vascular-targeted PDT.

Platelet-derived growth factor receptor β (PDGFRβ) is overexpressed on the pericytes of many types of tumors (Paulsson et al., 2009), suggesting that conjugation to PDGFRβ-binding molecules might deliver PS to tumor blood vessels. In previous works, several PDGFRβ-binding peptides were identified (Prakash et al., 2010; Askoxylakis et al., 2013; Marr et al., 2013). However, these peptides were limited by their low affinity (μM level) for PDGFRβ. Recently, Lindborg et al. (2011) identified numerous PDGFRβ-specific affibodies. Of these affibodies, Z02465 showed a nM affinity for human and mouse PDGFRβ. In addition, Z02465 did not bind PDGFRα, which is highly homologous to PDGFRβ. Due to its high affinity and specificity for PDGFRβ, affibody Z02465 and its derivative have been considered ideal tools for the delivery of anti-cancer drugs and contrast media. Tolmachev et al. (2014) found that affibody Z09591, a derivative of Z02465, was tumor homing in mice bearing glioma tumor grafts, which triggered our interests in developing this affibody as an anti-cancer drug carrier. In our previous work, we prepared the monomeric Z09591 affibody, designated Z_PDGFRβ_, and found that it showed PDGFRβ-dependent pericyte binding and thus increased the tumor uptake of fused anti-cancer proteins (Tao et al., 2017). These results suggested that the Z_PDGFRβ_ affibody might be used as a carrier of PS for pericyte-targeted PDT.

Since polymerization might improve the stability and avidity of the affibody (Kim et al., 2012), in this study, we first prepared the dimeric Z_PDGFRβ_ affibody and determined its PDGFRβ-dependent pericyte-binding and tumor-homing capabilities in mice bearing tumor grafts. Since IR700 dye is a soluble PS, we subsequently produced Z_IR700_ by conjugation of IR700 to Z_PDGFRβ_ and tested its photocytotoxicity and specificity in cultured cells. Finally, the antitumor effect of Z_IR700_-mediated PDT as a monotherapy or in combination with anti-cancer protein TRAIL was evaluated in mice bearing tumor grafts.

## Materials and methods

### Expression and purification of proteins

To produce the dimeric Z_PDGFRβ_ affibody, an artificial gene encoding Z09591 containing a unique cysteine (Tolmachev et al., 2014) with an additional HE-tag (Hofstrom et al., 2011) at its N-terminus was designed according to the codon usage preference of *E. coli*. The optimized gene was synthesized by GenScripts (Nanjing, China) followed by subcloning into the pQE30 plasmid with a deleted His-tag to construct the expressive plasmid pQE30-Z_PDGFRβ_. Expression of the Z_PDGFRβ_ affibody was induced by the addition of isopropyl β-D-thiogalactoside (IPTG, 0.1 mM) to a culture of *E. coli* M15 containing the pQE30-Z_PDGFRβ_ plasmid. After induction at 24 °C overnight, the culture was centrifuged at 7000 × *g* at 4 °C for 10 min. The pellets were resuspended in lysis buffer (50 mM phosphate (pH 8.0), 300 mM NaCl, and 5 mM imidazole) followed by processing 3–4 times in a high-pressure homogenizer (60–70 MPa). The recombinant Z_PDGFRβ_ affibody in the supernatant was recovered by using a Ni-NTA super flow column (Qiagen, Valencia, CA) according to the manual provided by the manufacturer. The recovered Z_PDGFRβ_ affibody was further dialyzed against phosphate-buffered saline (PBS, 10 mM Na_2_HPO_4_, 137 mM NaCl, 2.68 mM KCl, and 2 mM KH_2_PO_4_, pH 7.4) overnight and stored at −80 °C until further use. Sodium dodecyl sulfate-polyacrylamide gel electrophoresis (SDS-PAGE) and size-exclusion chromatography in the absence or presence of β-mercaptoethanol (2-ME) were used to estimate the purity and molecular weight of the Z_PDGFRβ_ affibody. The concentration of the gel for SDS-PAGE was 16%. In addition, a Superdex G-75 Increase 10/30 column (GE Healthcare, Anaheim, CA) was used for size-exclusion chromatography with PBS as the eluent. Protein concentration was measured by using a protein DC assay kit (Bio-Rad, Hercules, CA). Tumor necrosis factor-related apoptosis-inducing ligand (TRAIL) was prepared according to an earlier description (Li et al., 2016).

### PDGFRβ-binding assays

PDGFRβ-expressing pericytes were incubated with FAM- or IR700-labeled Z_PDGFRβ_ affibody at room temperature for 1 h. After two washes with PBS, the cells were analyzed by using a flow cytometer. To block the binding of Z_PDGFRβ_ affibody to PDGFRβ, cells were pre-incubated with a goat anti-human PDGFRβ antibody for 1 h before the addition of the Z_PDGFRβ_ affibody. In addition, PDGFRβ-binding of the Z_PDGFRβ_ affibody was further determined by using protein interaction analysis performed on an OpenSPR system (Nicoya Life Sciences Inc., Kitchener, Canada) (McGurn et al., 2016). Briefly, PDGFRβ-Fc (R&D, MN) was immobilized on the COOH-sensor chips, and solutions containing increasing concentrations of the Z_PDGFRβ_ affibody were introduced onto the chip, followed by surface plasmon resonance analysis on the OpenSPR system. The kinetic constants, including the association constant (*K*_a_), dissociation constant (*K*_d_) and affinity (KD, KD = *K*_d_/*K*_a_), were calculated using software according to a 1:1 binding model.

### Bio-distribution assay

Z_PDGFRβ_ affibody was labeled with CF™ 750 succinimidyl ester (CF750) or 5(6)-carboxyfluorescein (FAM) according to our previous description (Wei et al., 2015). Briefly, the pH value of the Z_PDGFRβ_ affibody (1.5 mg/ml) solution was adjusted to 8.0 by the addition of 1 M NaHCO_3_. The fluorescent dye dissolved in dimethyl sulfoxide (DMSO) was added to the Z_PDGFRβ_ affibody at a 6:1 (for CF750) or 20:1 (for FAM) molar ratio of dye to protein. After reaction at room temperature for 1 h in darkness, the mixture was dialyzed against PBS overnight. To monitor the tumor uptake of the Z_PDGFRβ_ affibody, a single dose (3.2 mg/kg) of the CF750-labeled Z_PDGFRβ_ affibody was intravenously injected into mice bearing LS174T tumor xenografts, followed by dynamic scanning with SPECTRAL Lago and Lago X Imaging Systems (Spectral, Tucson, AZ). At the end of this experiment, the organs/tissues of these mice were removed and scanned simultaneously. To determine the cellular distribution, the FAM-labeled Z_PDGFRβ_ affibody was intravenously injected into the mice. Subsequently, the tumor grafts were removed at different times post-injection and sectioned under freezing conditions. The nuclei were visualized by using DAPI. In addition, the FAM-labeled Z_PDGFRβ_ affibody was co-localized with CD31, PDGFRβ or NG2 by using immunofluorescence.

### Cell culture

Cell lines, including human colorectal cancer cells (LS174T), human umbilical vein-derived endothelial cells (ECs), and human uterine-derived smooth muscle cells (SMCs) were purchased from American Type Culture Collection (ATCC, Manassas, VA) and cultured in DMEM containing 10% fetal bovine serum, 2 mM L-glutamine, 100 U/ml penicillin, and 100 μg/ml streptomycin. Primary human brain vascular pericytes (PCs) were obtained from ScienCell (CA) and cultured in their specific media. All the cells were cultured at 37 °C in a 5% CO_2_ humidified atmosphere.

### Cytotoxicity assay

Cells (1 × 10^4^) were inoculated in the wells of a 96-well plate and cultured overnight. Subsequently, proteins diluted with media were added to the cells at different concentrations (0–10 μM). The same volume of PBS was used as a control. After treatment for 16–18 h, the viable cells were determined by the addition of Cell Counting Kit-8 (CCK-8) solution (Dojindo, Japan). The viability of PBS-treated cells was considered 100%.

### Expression of biomarkers in cells and tissues

Expression of PDGFRβ, NG2, or α-SMA in cells was detected by flow cytometry. Cells were incubated with primary antibody for 1 h at room temperature followed by further incubation with secondary antibody for 30 min (if needed) prior to analysis with a flow cytometer. For immunofluorescence, the tumor grafts were sectioned under freezing conditions immediately after removal from the mice. To localize the expression of biomarkers in tumor blood vessels, tumor tissues were co-stained with antibody against PDGFRβ, NG2, or α-SMA and antibody against CD31, followed by observation under a fluorescence microscope. The primary antibodies included rat anti-mouse CD31 and PE-mouse anti-human PDGFRβ (Biolegend, San Diego, CA), goat anti-human PDGFRβ (R&D, MN), and rabbit anti-human PDGFRβ, rabbit anti-human NG2, and rabbit anti-human α-SMA (Abcam, Burlingame, CA). Secondary antibodies included donkey anti-rabbit IgG (DyLight 488) and goat anti-rat IgG (DyLight 550) (Abcam, Burlingame, CA). An isotype antibody was used as a control.

### Conjugation of the photosensitizer to the Z_PDGFRβ_ affibody

The photosensitizer IRDye 700DX N-hydroxysuccinimide ester (IR700) was obtained from LI-COR Biosciences (Lincoln, NE). Conjugation was performed as described by Mitsunaga et al. (2011) with some modifications. Briefly, IR700 dissolved in DMSO was mixed with the Z_PDGFRβ_ affibody (1.5 mg/ml, pH 8.0) at a molar ratio of 2:1 (dye to protein) followed by incubation at room temperature for 1 h in darkness. The conjugation of IR700 to the Z_PDGFRβ_ affibody was verified by SDS-PAGE followed by scanning the gel with Odyssey CLx (LI-COR Biosciences, Lincoln, NE). The conjugate was designated Z_IR700_.

### *In vitro* PDT

Cells (1 × 10^4^/well) were inoculated in 96-well plates. After incubation overnight, Z_IR700_ (4 μM IR700 equivalent) was added to the wells followed by two washes with PBS 1 h later. Subsequently, 100 μl medium without phenol red was added to the wells, and the cells were illuminated at a fluence rate of 16 mW/cm^2^ for a total dose of 10 J/cm^2^ with a laser at a wavelength of 690 nm. The viability of the cells was examined by using a live/dead BacLight bacterial viability kit (Invitrogen, Carlsbad, CA) (Shirasu et al., 2014). Non-illuminated cells were used as a control.

Dichlorodihydrofluorescein diacetate (DCFH) and singlet oxygen sensor green (SOSG) were used to detect hydroxyl radicals (Setsukinai et al., 2003) and singlet oxygen (Gollmer et al., 2011; van Driel et al., 2016), respectively. DCFH or SOSG (10 μM) plus Z_IR700_ were pre-incubated with cells for 1 h followed by two washes with PBS. Subsequently, 100 μl medium without phenol red was added to the cells followed by illumination. Approximately 1–2 h later, the fluorescence was measured using a fluorescence microplate reader (λ_exc_ = 488 nm). Simultaneously, the cells were observed under a fluorescence microscope after the nuclei were visualized using Hoechst.

### *In vivo* PDT

Approximately, 2 × 10^6^ LS174T cells were subcutaneously implanted into the right hind leg of BALB/c nude mice (*n* = 3). Once the tumor size reached 150 mm^3^, Z_IR700_ (0.8 mg/kg Z_PDGFRβ_ affibody equivalent) was intravenously injected into the mice. Approximately 4 h later, the tumor grafts were illuminated at a fluence rate of 100 mW/cm^2^ for a total dose of 120 J/cm^2^. The longitudinal (*L*) and transverse (*W*) diameters of the tumor grafts were measured every day. In addition, the tumor volume (*V*) was calculated according to the following formula: *V* = *L* × *W*^2^/2. At the end of experiment, all tumor grafts were removed and weighed.

For histochemistry, tumor grafts were removed at different times post-illumination. The paraffin-sectioned tumor tissues were stained by H&E. To assess the leakage of tumor blood vessels, fluorescein isothiocyanate (FITC)-labeled dextran (70 kDa, Sigma, St. Louis, MO) was intravenously injected into mice bearing LS174T tumor grafts at different times post-illumination (0–8 h). The dextran was allowed to circulate for 20 min. To perfuse the mouse, 60 ml PBS was injected from the ventriculus sinister and drained from the right atrium. Subsequently, the tumor grafts were removed and sectioned (50 μm) under freezing conditions. Blood vessels were visualized by immunofluorescence with anti-mouse CD31. In addition, the nuclei were visualized with DAPI. Finally, the tumor tissues were scanned using a multiphoton laser scanning confocal microscope (Nikon, Tokyo, Japan). To illustrate hypoxia in tumor grafts, expression of hypoxia-inducible factor 1α (HIF1α) was detected by using a rabbit anti-human HIF1α antibody (Novus Biologicals, San Diego, CA).

### Combination therapy of Z_IR700_-mediated PDT and TRAIL

Selective cytotoxicity of TRAIL in tumor cells highlighted its potential as a candidate drug for cancer therapy (Holland, 2013). The increase of vessel permeability induced by vascular targeted PDT triggered our interest on the combination therapy of Z_IR700_-mediated PDT and TRAIL. To determine whether Z_IR700_-mediated PDT would increase the tumor uptake of TRAIL, a single dose (10 mg/kg) of FAM-labeled TRAIL was intravenously injected into the mice bearing LS174T tumor grafts prior to illumination. The tumor grafts were removed 1 h post-illumination and sectioned under freezing conditions. After the tumor tissues were stained with antibody against mouse CD31, they were observed under a fluorescence microscope. Since TRAIL induces apoptosis in tumor cells, the increase in the tumor uptake of TRAIL was also reflected by an increase in apoptotic cells in the tumor tissues. To visualize the apoptotic cells, tumor grafts were removed 16 h post-illumination and sectioned under freezing conditions, followed by TdT-mediated dUTP nick-end labeling (TUNEL).

To compare the antitumor effect of different regimens, mice (*n* = 6–7) bearing LS174T tumor grafts were treated with PBS, TRAIL (10 mg/kg), Z_IR700_ (0.8 mg/kg), and Z_IR700_ (0.8 mg/kg)/TRAIL (10 mg/kg). Additionally, mice treated with Z_IR700_ or Z_IR700_/TRAIL were illuminated at a fluence rate of 100 mW/cm^2^ for a total dose of 120 J/cm^2^ after each injection. The tumor volume was measured every day. At the end of the experiment, all tumor grafts were removed and weighed.

### Statistical analysis

SPSS software (version 20) was used for one-way analysis of variance (ANOVA) for multiple comparisons. The significance level was defined as *p* < .05. The results are expressed as the mean ± standard deviation (SD).

## Results

### PDGFRβ-positive cells are predominantly located with the mural cells of tumor blood vessels

**Figure 1.**
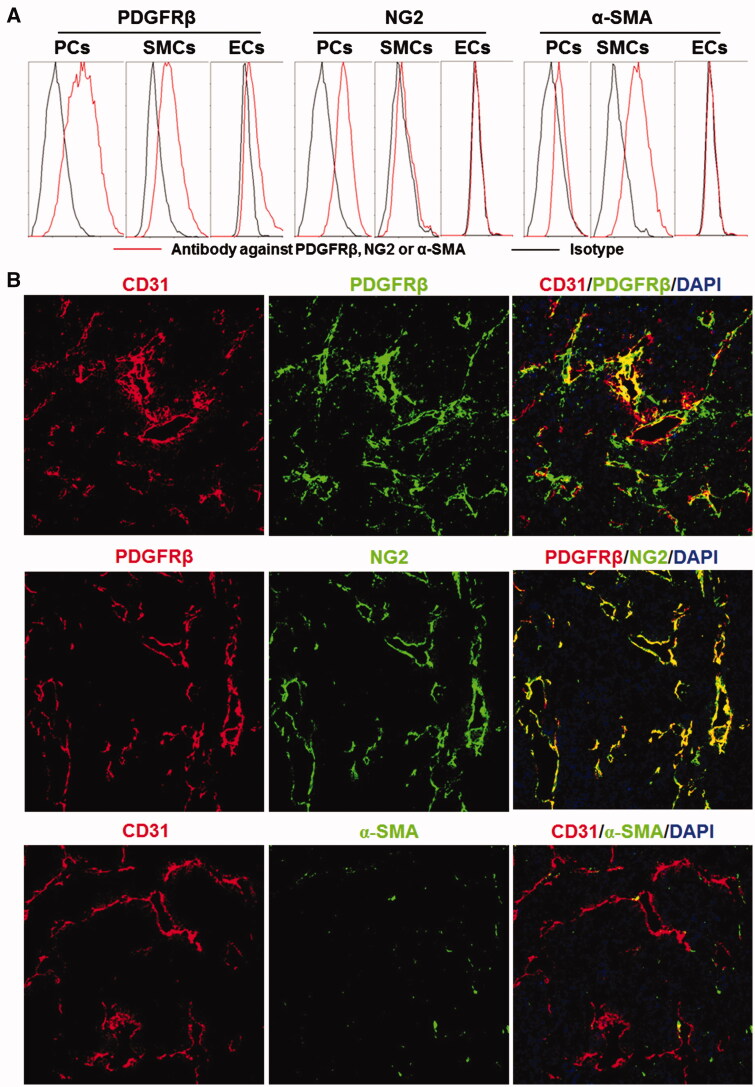
Expression of surface markers on mural cells. (A) Expression of PDGFRβ, NG2, and α-SMA in pericytes (PCs), smooth muscle cells (SMCs), and endothelial cells (ECs). (B) Co-localization of PDGFRβ, NG2, or α-SMA with CD31 in tumor tissues derived from LS174T tumor grafts. The nuclei were visualized by DAPI staining. Original magnification 200×.

Tumor blood vessels can be composed of endothelial cells (ECs), pericytes (PCs), and smooth muscle cells (SMCs). Endothelial cells can be characterized by CD31 expression. However, there is no exclusive marker for pericytes or smooth muscle cells. Consequently, the identification of pericytes and smooth muscle cells depends on both cell surface markers and cell location (Geevarghese & Herman, 2014). As shown in Figure 1(A), flow cytometry demonstrated that the PDGFRβ-positive (PDGFRβ^+^) rates of cultured pericytes and smooth muscle cells were 72.9 and 47.6% compared to 10.1% for that of endothelial cells, indicating that PDGFRβ was predominantly expressed on pericytes and smooth muscle cells. Expression of NG2 was detectable in 66.5% of pericytes compared to less than 10% for that of smooth muscle cells and endothelial cells. Approximately 70% of smooth muscle cells were α-SMA positive (α-SMA^+^), whereas both pericytes and endothelial cells were α-SMA negative (α-SMA^−^, positive rate <8%). These results demonstrated that pericytes were PDGFRβ^+^NG2^+^α-SMA^−^, whereas smooth muscle cells were PDGFRβ^+^NG2^−^α-SMA^+^. Consequently, of these three cells, NG2 might be considered an exclusive marker for pericytes. As shown in Figure 1(B), most PDGFRβ^+^ cells in LS174T tumor tissues were proximate to CD31-positive (CD31^+^) endothelial cells, suggesting that these PDGFRβ^+^ cells are mural cells. Moreover, the distribution profile of PDGFRβ is identical to that of NG2, whereas few α-SMA^+^ cells were observed in tumor tissues, indicating that among the mural cells of tumor blood vessels, PDGFRβ^+^ cells were predominantly pericytes, not smooth muscle cells.

### Z_PDGFRβ_ affibody binds PDGFRβ with high affinity

**Figure 2.**
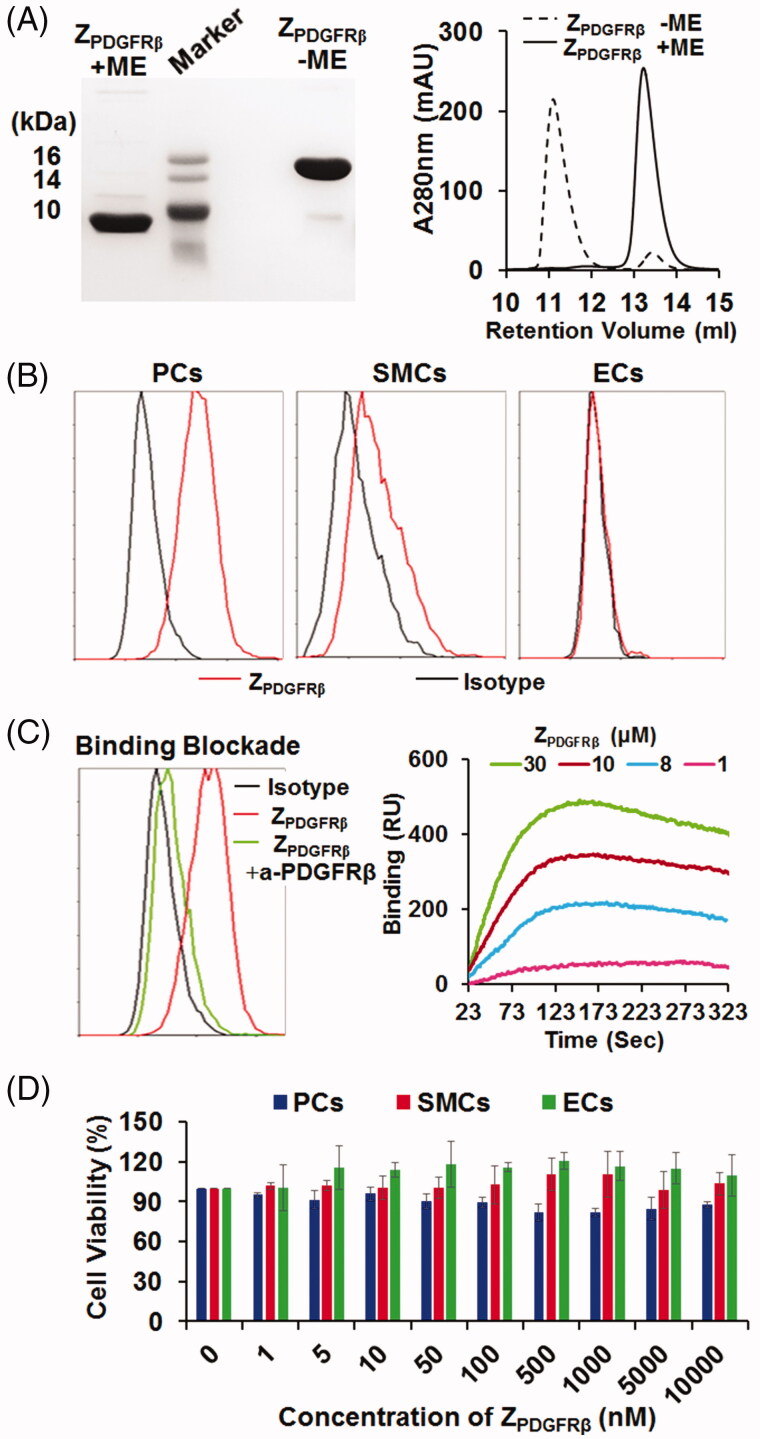
Preparation and characterization of the Z_PDGFRβ_ affibody. (A) SDS-PAGE and size-exclusion chromatography of the purified Z_PDGFRβ_ affibody in the presence or absence of 2-ME. (B) Binding of the Z_PDGFRβ_ affibody to pericytes (PCs), smooth muscle cells (SMCs), and endothelial cells (ECs) as analyzed by flow cytometry. (C) Inhibition of pericyte binding of the Z_PDGFRβ_ affibody by the PDGFRβ-specific antibody (left) and the PDGFRβ-binding of the Z_PDGFRβ_ affibody analyzed using the OpenSPR system (right). (D) Cytotoxicity of the Z_PDGFRβ_ affibody in pericytes (PCs), smooth muscle cells (SMCs), and endothelial cells (ECs).

As shown in Figure 2(A), purified Z_PDGFRβ_ affibody was visualized as a single band on an SDS-PAGE gel. The molecular weight of the Z_PDGFRβ_ affibody under natural conditions (in the absence of 2-ME) was approximately 2-fold (∼15 kDa versus 7 kDa) that of the Z_PDGFRβ_ affibody under reductive conditions (in the presence of 2-ME), indicating that the Z_PDGFRβ_ affibody forms disulfide bond-containing dimers under natural conditions. Size-exclusion chromatography further revealed that over 90% of the Z_PDGFRβ_ affibody exists as a dimer under natural conditions. The binding rate of the Z_PDGFRβ_ affibody to pericytes was 71%, while that of the Z_PDGFRβ_ affibody to smooth muscle cells was 28.4%. In addition, the binding of the Z_PDGFRβ_ affibody to endothelial cells was undetectable (Figure 2(B)). The binding rate of the Z_PDGFRβ_ affibody to pericytes, smooth muscle cells, and endothelial cells was closely related to the expression level of PDGFRβ in these cells. Moreover, the binding of the Z_PDGFRβ_ affibody to pericytes was drastically (from 70% to 10%) reduced by pre-incubation of the cells with a blocking antibody against PDGFRβ (Figure 2(C)), indicating that the Z_PDGFRβ_ affibody specifically binds PDGFRβ. Protein–protein interaction analysis demonstrated that the Z_PDGFRβ_ affibody binds PDGFRβ with an affinity of 0.9 nM. However, even treatment with 10 μM of the Z_PDGFRβ_ affibody overnight did not reduce the viability of these three cells (Figure 2(D)). These results indicated that the Z_PDGFRβ_ affibody binds PDGFRβ with a high affinity but is not cytotoxic in the tested PDGFRβ-positive cells.

### Z_PDGFRβ_ affibody is tumor homing

**Figure 3.**
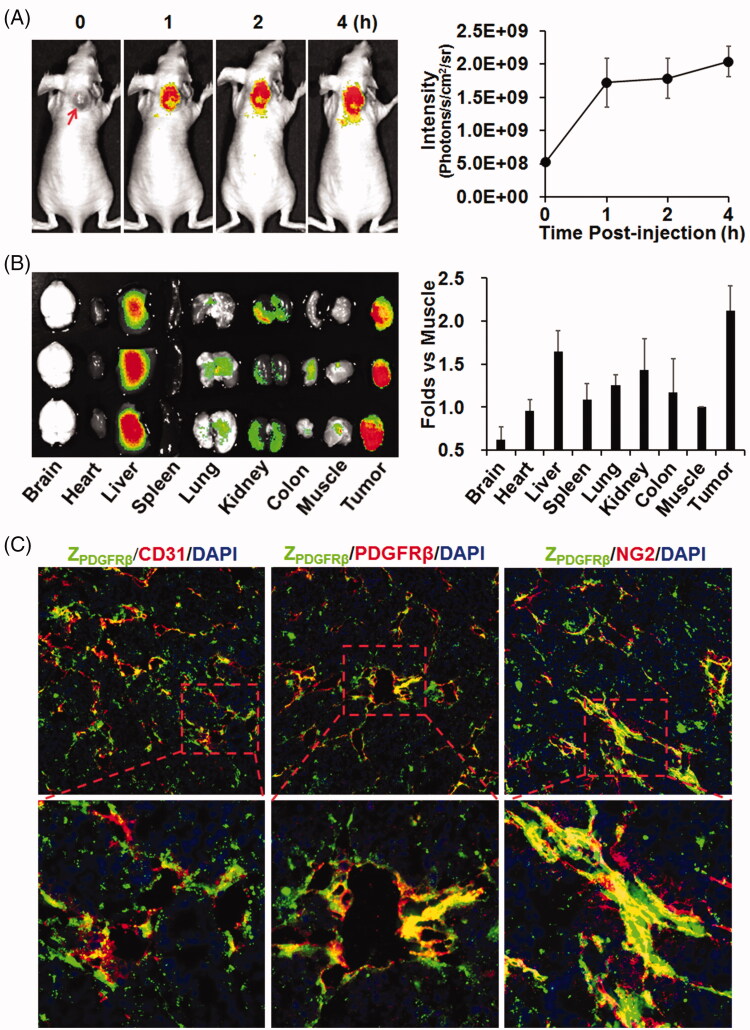
Tumor-homing characteristics of the Z_PDGFRβ_ affibody. (A) Tumor uptake of the Z_PDGFRβ_ affibody. CF750-labeled Z_PDGFRβ_ affibody was intravenously injected into mice (*n* = 3) bearing subcutaneous LS174T tumor grafts (arrow indicated), followed by scanning with SPECTRAL Lago and Lago X Imaging Systems at different times (0–4 h) post-injection. (B) Bio-distribution of the Z_PDGFRβ_ affibody in mice bearing LS174T tumor grafts. Mice (*n* = 3) injected with CF750-labeled Z_PDGFRβ_ affibody were sacrificed at 4 h post-injection for optical imaging of organs/tissues. (C) Co-localization of the Z_PDGFRβ_ affibody with CD31, PDGFRβ, or NG2 in tumor tissues. FAM-labeled Z_PDGFRβ_ affibody was intravenously injected into mice bearing LS174T tumor grafts. Tumor grafts were removed and sectioned under freezing conditions, followed by staining with antibody against CD31, PDGFRβ, or NG2. The nuclei were visualized by DAPI staining. Original magnification 200×.

To test its tumor-homing characteristics, CF750-labeled Z_PDGFRβ_ affibody was intravenously injected into mice bearing LS174T tumor grafts followed by dynamic optical imaging. As shown in Figure 3(A), optical images demonstrated that the Z_PDGFRβ_ affibody accumulated in subcutaneous tumor grafts from 1 h post-injection and persisted till at least 4 h. Organ/tissue scanning at 4 h post-injection confirmed the accumulation of the Z_PDGFRβ_ affibody in tumor grafts. Notably, the uptake of the Z_PDGFRβ_ affibody by tumor grafts was even higher than that of the Z_PDGFRβ_ affibody by livers and kidneys (Figure 3(B)). These results indicated that the Z_PDGFRβ_ affibody is tumor homing. Moreover, cellular localization analysis demonstrated that FAM-labeled Z_PDGFRβ_ affibody co-localized well with PDGFRβ and with NG2 (Figure 3(C) and Supplementary Figure S1), suggesting that the Z_PDGFRβ_ affibody was predominantly distributed on pericytes in the mural of tumor blood vessels.

### Z_IR700_-mediated PDT specifically kills PDGFRβ-expressing cells

**Figure 4.**
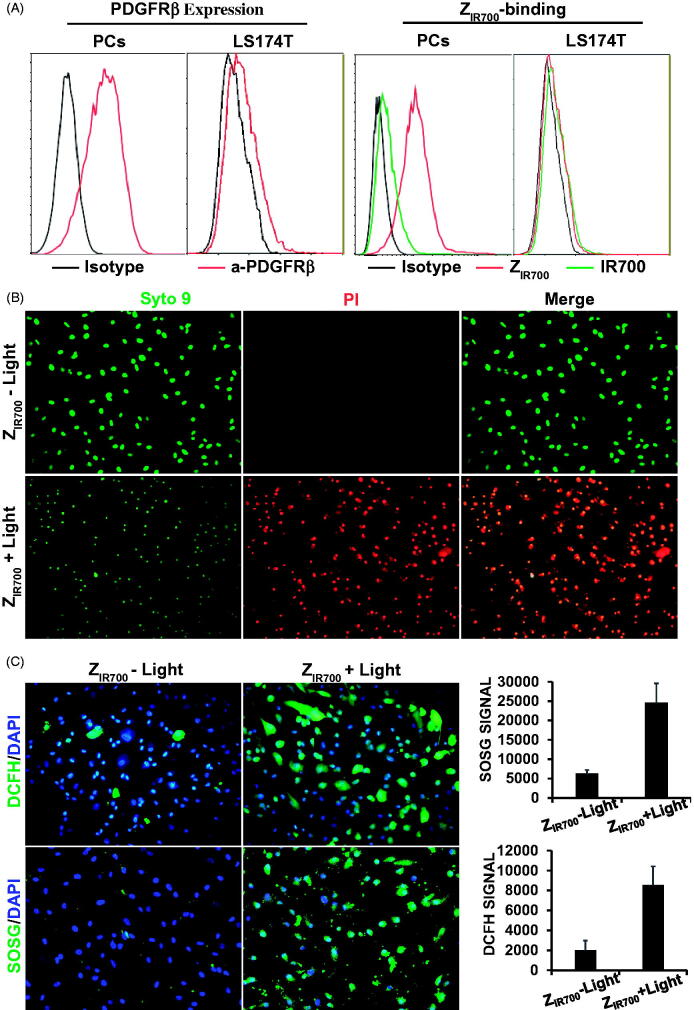
Z_IR700_-mediated PDT kills PDGFRβ-expressing cells *in vitro*. (A) Flow cytometry analysis of PDGFRβ expression (left) and Z_IR700_ binding (right) to pericytes (PCs) and LS174T tumor cells. (B) Cytotoxicity of Z_IR700_ in PDGFRβ-expressing pericytes under different conditions. Cells were pre-incubated with Z_IR700_ for 1 h followed by washing with PBS and illumination with (Z_IR700_+Light) or without (Z_IR700_-Light) laser. The live/dead cells were visualized by SYTO9/PI staining. Original magnification 200×. (C) Production of ROS in pericytes treated with Z_IR700_-mediated PDT. Cells were incubated with DCFH or SOSG during Z_IR700_-mediated PDT and were observed under a fluorescence microscope (left) or measured with a fluorescence microplate reader (right). Original magnification 200×.

To evaluate the specificity of Z_IR700_-mediated PDT, the photocytotoxicity of Z_IR700_ was determined in PDGFRβ^+^ and PDGFRβ^−^ cells. As shown in Figure 4(A), flow cytometry demonstrated that the 73% of the pericytes were PDGFRβ^+^ and that 8% of the LS174T tumor cells were PDGFRβ^+^. The binding rate of Z_IR700_ to pericytes was 75%, whereas its binding rate to LS174T tumor cells was 4%. The free IR700 dye showed little (<8%) binding to both cells. Illumination induced over 90% death of PDGFRβ^+^ pericytes, which were pre-incubated with Z_IR700_ and washed prior to illumination (Figure 4(B)). However, illumination did not induce obvious cell death in PDGFRβ^−^ LS174T tumor cells under the same conditions (Supplementary Figure S2(A)). After incubation of PDGFRβ^+^ pericytes with free IR700 dye, illumination only induced obvious death of unwashed cells (Supplementary Figure S2(B)). These results demonstrated that the binding of Z_IR700_ to pericytes was mediated by the Z_PDGFRβ_ affibody, which is specific for PDGFRβ. Figure 4(C) demonstrates that reactive oxygen species (ROS), including hydroxyl radicals (DCFH signal) and singlet oxygen (SOSG signal), accumulated in pericytes treated with Z_IR700_-mediated PDT. Since ROS contribute to the phototoxicity of PS (Dąbrowski, 2017), these results indicated that Z_IR700_-mediated PDT killed PDGFRβ^+^ pericytes by ROS-mediated phototoxicity.

### Z_IR700_-mediated PDT suppresses tumor growth by damaging blood vessels

**Figure 5.**
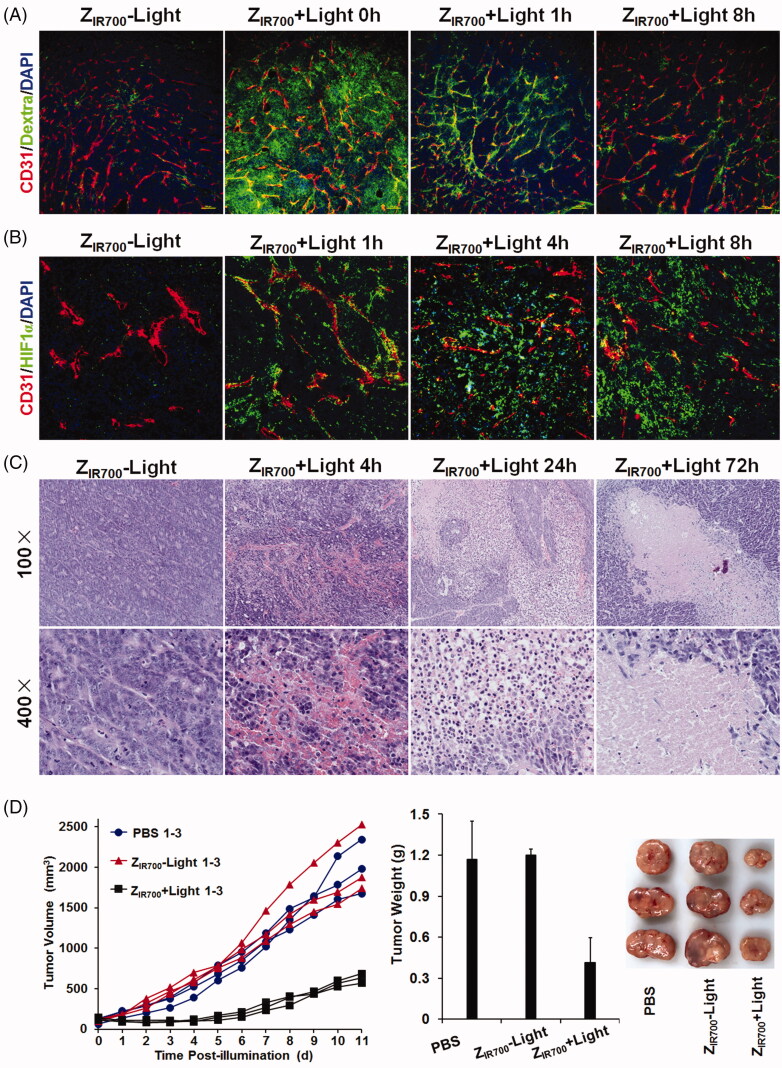
Z_IR700_-mediated PDT suppresses LS174T tumor growth in mice. (A) Permeability of tumor blood vessels visualized by the leakage of dextran. After intravenous injection of Z_IR700_ followed by illumination (Z_IR700_ + Light) or not (Z_IR700_−Light), mice bearing LS174T tumor grafts were intravenously injected with FITC-dextran at different times (0–8 h) post-illumination. Approximately 20 min later, the tumor grafts were removed and sectioned under freezing conditions, followed by visualization of the blood vessels with antibody against CD31. DAPI was used to visualize the nuclei. Original magnification 100×. (B) Expression of HIF1α in tumor tissues at different times (1–8 h) post-illumination. Original magnification 200×. (C) H&E staining of tumor tissues derived from tumor grafts at different times (0–72 h) post-illumination. (D) Tumor growth suppression by Z_IR700_-mediated PDT. Mice (*n* = 3) bearing LS174T tumor grafts were intravenously injected with Z_IR700_, followed by illumination (Z_IR700_+Light) or not (Z_IR700_-Light) at day 0 and day 3, respectively. Mice in the control group were treated with PBS. The tumor volumes were measured every day, and the (1–3) growth curves of mice in each group are shown. At the end of this experiment, all tumor grafts were removed and weighed.

Since Z_IR700_-mediated PDT induced the death of pericytes *in vitro*, it might damage tumor blood vessels, thus increasing their permeability once *in vivo* PDT is performed. To determine the change in the permeability of tumor blood vessels, FITC-labeled dextran was intravenously injected into mice bearing LS174T tumor grafts at different times post-illumination. As shown in Figure 5(A) and Supplementary Figure S3, little dextran was observed in tumor tissues derived from mice injected with Z_IR700_ without illumination. However, when injected at the beginning of illumination (0 h post-illumination), dextran was observed in a wide range of tumor tissues derived from mice treated with Z_IR700_-mediated PDT, indicating the leakage of tumor blood vessels. When dextran was injected at 1 or 8 h post-illumination, although the amount of dextran decreased drastically, dextran was still observed in tumor blood vessels and neighboring tissues. These results demonstrated that Z_IR700_-mediated PDT increases the permeability of tumor blood vessels.

The level of HIF1α reflects tissue hypoxia. As shown in Figure 5(B), few cells were HIF1α expressive in LS174T tumor grafts from mice injected with Z_IR700_. Z_IR700_-mediated PDT increased the number of HIF1α-expressing cells in tumor grafts over time, indicating the intensification of hypoxia in tumor grafts. In addition, a great number of red blood cells were observed in tumor tissues collected within 8 h post-illumination. Blood clots were visible in tumor tissues collected between 1 and 4 h post-illumination (Figure 5(C)). These results indicated that Z_IR700_-mediated PDT damages tumor blood vessels and induces hemorrhage and thrombosis, which would impair the delivery function of tumor blood vessels, thus intensifying hypoxia in tumor grafts. Approximately 24 h post-illumination, a wide range of tumor tissues became non-coherent. In addition, most cells in non-coherent tumor tissues disappeared at 72 h post-illumination (Figure 5(C)). Since hypoxia would induce necrosis/apoptosis of tumor cells, these results demonstrated that Z_IR700_-mediated PDT damaged tumor blood vessels, thereby inducing tumor destruction by the intensification of tissue hypoxia.

Moreover, the growth of LS174T tumor grafts in mice treated with Z_IR700_-mediated PDT was much slower than that in mice injected with Z_IR700_ without illumination (Figure 5(D) and Supplementary Figure S4). At the end of this experiment, the average tumor mass of mice treated with Z_IR700_-mediated PDT was 0.41 ± 0.04 g, compared to 1.20 ± 0.18 g of mice injected with Z_IR700_ without illumination and 1.17 ± 0.28 g of PBS-treated mice. These results demonstrated that Z_IR700_-mediated PDT significantly (*p* < .05) suppresses tumor growth in mice.

### Z_IR700_-mediated PDT and TRAIL combination therapy yields a better antitumor effect

**Figure 6.**
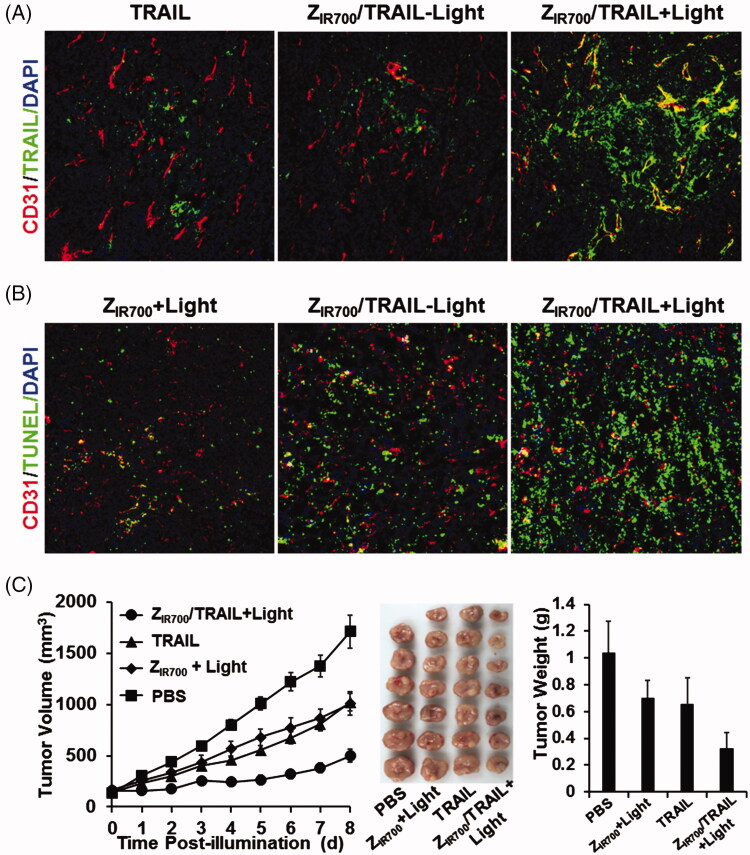
Z_IR700_-mediated PDT and TRAIL combination therapy leads to a better antitumor effect. (A) Z_IR700_-mediated PDT increases the tumor uptake of TRAIL. Mice bearing LS174T tumor grafts were treated with FAM-labeled TRAIL (TRAIL) or Z_IR700_ combined with TRAIL followed (Z_IR700_/TRAIL + Light) or not followed by (Z_IR700_/TRAIL-Light) illumination. The tumor grafts were removed and sectioned under freezing conditions 1 h post-injection of TRAIL. The blood vessels were visualized with antibody against CD31. The nuclei were visualized by DAPI staining. Original magnification 200×. (B) Z_IR700_-mediated PDT and TRAIL combination therapy induced stronger apoptosis. Mice bearing LS174T tumor grafts were treated with Z_IR700_-mediated PDT alone (Z_IR700_+Light) or Z_IR700_ combined with TRAIL followed (Z_IR700_/TRAIL + Light) or not followed (Z_IR700_/TRAIL-Light) by illumination. Tumor grafts were removed 16 h post-injection of TRAIL and sectioned under freezing conditions. TUNEL was used to visualize apoptotic cells in tumor tissues. Original magnification 200×. (C) Z_IR700_-mediated PDT and TRAIL combination therapy shows greater tumor growth suppression. Mice (*n* = 6–7) were treated with TRAIL or Z_IR700_-mediated PDT (Z_IR700_+Light) or TRAIL and Z_IR700_-mediated PDT combination therapy (Z_IR700_/TRAIL + Light). PBS was used as a control.

Since Z_IR700_-mediated PDT increased the permeability of tumor blood vessels, we attempted to investigate whether it would increase the tumor uptake of the protein drug TRAIL, thereby demonstrating a better antitumor effect. To monitor the tumor uptake, FAM-labeled TRAIL was intravenously injected into the mice at the beginning of illumination during Z_IR700_-mediated PDT. As shown in Figure 6(A), Z_IR700_-mediated PDT obviously increased the tumor uptake of TRAIL. Since TRAIL could induce apoptosis in tumor cells, the numbers of apoptotic cells were used to reflect the uptake of TRAIL under different conditions. As shown in Figure 6(B), compared to the monotherapy based on Z_IR700_-mediated PDT or TRAIL, combination therapy of Z_IR700_-mediated PDT and TRAIL induced greater apoptosis in tumor cells. Accordingly, the growth of tumor grafts treated with combination therapy was slower than that of tumor grafts treated with monotherapy. At the end of this experiment, the average tumor mass after combination therapy was 0.33 ± 0.11 g, compared to 0.65 ± 0.20 g (for TRAIL) and 0.69 ± 0.13 g (Z_IR700_-mediated PDT) after monotherapy. These results demonstrated that Z_IR700_-mediated PDT could increase tumor uptake of TRAIL, thus leading to greater tumor growth suppression.

## Discussion

Conventional tumor vascular-targeted PDT is achieved by the injection of free PS followed by illumination within a short time (several minutes), when the majority of PS is retained in the blood vessels (Krzykawska-Serda et al., 2014; Azzouzi et al., 2017). The short drug (PS administration)-to-light (illumination) interval (DLI) and non-specific accumulation of PS would definitely limit the application of passive PS-delivery-based vascular-targeted PDT in the clinic. In recent years, increasing attention has been paid to active PS-delivery-based vascular-targeted PDT (Kamarulzaman et al., 2011). Theoretically, as the major vascular cells of tumors, both endothelial cells and pericytes could be considered target cells for PS delivery. Endothelial cell-targeted PS delivery has been achieved by the conjugation of PS to VEGFR- or αvβ3 integrin-binding molecules (Thomas et al., 2010; Srivatsan et al., 2011). However, tumor vascular-targeted PDT based on pericyte-targeted PS delivery has not been reported.

Endothelial cells compose the endothelium, whereas pericytes cover the endothelial tubule. In tumor blood vessels, endothelial cells irregularly line the walls of the vessel, which allows delivery of PS to pericytes (Chang et al., 2013; Geevarghese & Herman, 2014). However, the pericytes in normal blood vessels are shielded by the intact endothelial-cell barrier. Consequently, compared to the endothelial cell-targeted strategy, pericyte-targeted PS delivery involves a lower risk of accumulation of PS in normal tissues. Owing to the high expression of PDGFRβ in tumor-associated pericytes (Paulsson et al., 2009) and the high affinity and specificity of Z_PDGFRβ_ affibody for PDGFRβ (Lindborg et al., 2011; Tao et al., 2017), pericyte-targeted delivery can be achieved by the conjugation of PS to the Z_PDGFRβ_ affibody. It is noteworthy that among vascular cells, smooth muscle cells also express PDGFRβ. Unlike the normal mature blood vessels, most tumor blood vessels are immature, with cellular abnormalities (Baluk et al., 2005). In fact, few smooth muscle cells were observed in tumor blood vessels (Figure 1(B)). Consequently, pericytes are major PDGFRβ^+^ mural cells in tumor grafts, thus conjugation to the Z_PDGFRβ_ affibody would deliver PS to pericytes.

Due to the ease of purification and reduced hepatic accumulation of the HE-tagged affibody (Hofstrom et al., 2011), a HE-tag, not a His-tag, was introduced at the N-terminus of Z09591 to produce the Z_PDGFRβ_ affibody. In addition, the Z_PDGFRβ_ affibody was designed to form a dimer upon the addition of a cysteine residue at the C-terminus. As expected, most Z_PDGFRβ_ affibodies formed dimers under natural conditions (Figure 2(B)). In addition, the affinity (0.9 nM) of the dimeric Z_PDGFRβ_ affibody for human PDGFRβ (Figure 2(C)) was higher than that (4.5 nM) of the monomeric Z_PDGFRβ_ affibody (Tao et al., 2017). In mice bearing LS174T tumor grafts, accumulation of the dimeric Z_PDGFRβ_ affibody in tumor grafts was higher than that in the liver or the kidney at 4 h post-injection (Figure 3(A,B)), indicating the tumor-homing characteristic of the Z_PDGFRβ_ affibody. Among vascular cells, NG2 is exclusively expressed on pericytes (Figure 1(A)). The Z_PDGFRβ_ affibody co-localized well with NG2 in the mural cells of tumor blood vessels (Figure 3(C) and Supplementary Figure S1), demonstrating that it was predominantly distributed on pericytes in tumor blood vessels. Z_IR700_, the conjugate of the Z_PDGFRβ_ affibody and IR700, bound pericytes and induced obvious cell death *in vitro* after excitation by light (Figure 4(B) and Supplementary Figure S2(A)). Since pericytes are major mural cells, Z_IR700_-mediated PDT *in vivo* might damage them, thus increasing the permeability of tumor blood vessels.

To determine the PDT-induced increase in permeability, dextran (∼70 kDa) was intravenously injected into mice bearing LS174T tumor grafts at different times post-illumination. As shown in Figure 5(A), Z_IR700_-mediated PDT induced the leakage of dextran injected at the beginning (0 h) of illumination. Tumor uptake of TRAIL injected at the same time point was increased by Z_IR700_-mediated PDT (Figure 6(A)). In addition, numerous red blood cells were observed in tumor tissues collected at different times post-illumination (Figure 5(C)). These results demonstrated that Z_IR700_-mediated PDT rapidly damaged tumor blood vessels and induced hemorrhage. However, compared to tumor accumulation of the dextran injected at the beginning (0 h) of illumination, that of dextran injected at 1 and 8 h post-illumination decreased drastically (Figure 5(A)). However, HIF1α expression, reflecting hypoxia in tumor tissues, increased over time post-illumination (Figure 5(B)). Moreover, blood clots were observed in tumor tissues collected between 1 and 4 h post-illumination (Figure 5(C)). These results demonstrated that thrombosis, and even vessel occlusion, occurred after a short time post-illumination. Consequently, the delivery function of tumor blood vessels was impaired, and tissue hypoxia was intensified. It is known that intensified hypoxia can induce the necrosis/apoptosis of tumor cells (van Straten et al., 2017). In fact, tumor destruction was observed in tumor grafts collected at 24 and 72 h post-illumination (Figure 5(C)). Due to the inefficiency of ROS to kill bystander cells (Heukers et al., 2014), Z_IR700_-mediated pericyte-targeted PDT would not kill bystander LS174T tumor cells directly, suggesting that Z_IR700_-mediated PDT induced tumor destruction by intensifying hypoxia.

To improve the efficacy, the DLI for passive PS-delivery-based vascular-targeted PDT is usually shortened to several minutes. For example, long-term tumor suppression was observed when verteporfin was excited by light at 5 min post-injection. If the illumination was performed at 3 h post-injection of verteporfin, no apparent acute vascular responses were seen (Fingar et al., 1999). In this experiment, vascular responses and long-term tumor suppression were observed even when illumination was performed at 4 h post-injection of Z_IR700_ (Figures 5 and 6). These results suggested that DLI of the Z_PDGFRβ_ affibody-based active vascular-targeted PDT might be considerably longer than that of passive vascular-targeted PDT, which would drastically reduce the adverse effects of PS. Compared to the big (∼150 kDa) antibody, the small (∼6–7 kDa) affibody has the advantage of superior tumor penetration (Sexton et al., 2013). In addition, high yield (20–30 mg/l culture) of the small affibody in *E. coli* would reduce the cost of PDT. These results highlighted the potential of Z_PDGFRβ_ affibody-based vascular-targeted PDT for cancer therapy.

## Conclusions

Abnormal structures of tumor blood vessels make PDGFRβ-positive pericytes attractive target cells for drug delivery. Due to the high affinity and specificity for PDGFRβ, the Z_PDGFRβ_ affibody was predominantly distributed on PDGFRβ^+^ pericytes and accumulated in tumor grafts. The conjugate of the Z_PDGFRβ_ affibody and IR700, i.e. Z_IR700_, specifically killed pericytes once excited by light. Z_IR700_-mediated PDT damaged tumor blood vessels thus induced tumor destruction in mice. In addition, Z_IR700_-mediated PDT enhanced the antitumor effect of TRAIL by increasing its tumor uptake. Taken together, these results demonstrate that the Z_PDGFRβ_ affibody could be used as a PS carrier for active vascular-targeted PDT as a monotherapy as well as combination therapy for cancers with rich pericytes.

## Supplementary Material

IDRD_Lu_et_al_Supplemental_Content.docx
