# Supplementary material for: PDGFRβ-specific affibody-directed delivery of a photosensitizer, IR700, is efficient for vascular-targeted photodynamic therapy of colorectal cancer
Source: Drug Deliv. 2017 Nov 28;24(1):1818–30. doi: 10.1080/10717544.2017.1407011 (PMC8240977; doi:10.1080/10717544.2017.1407011)
Supplement: IDRD_Lu_et_al_Supplemental_Content.docx [file IDRD_A_1407011_SM3452.docx]

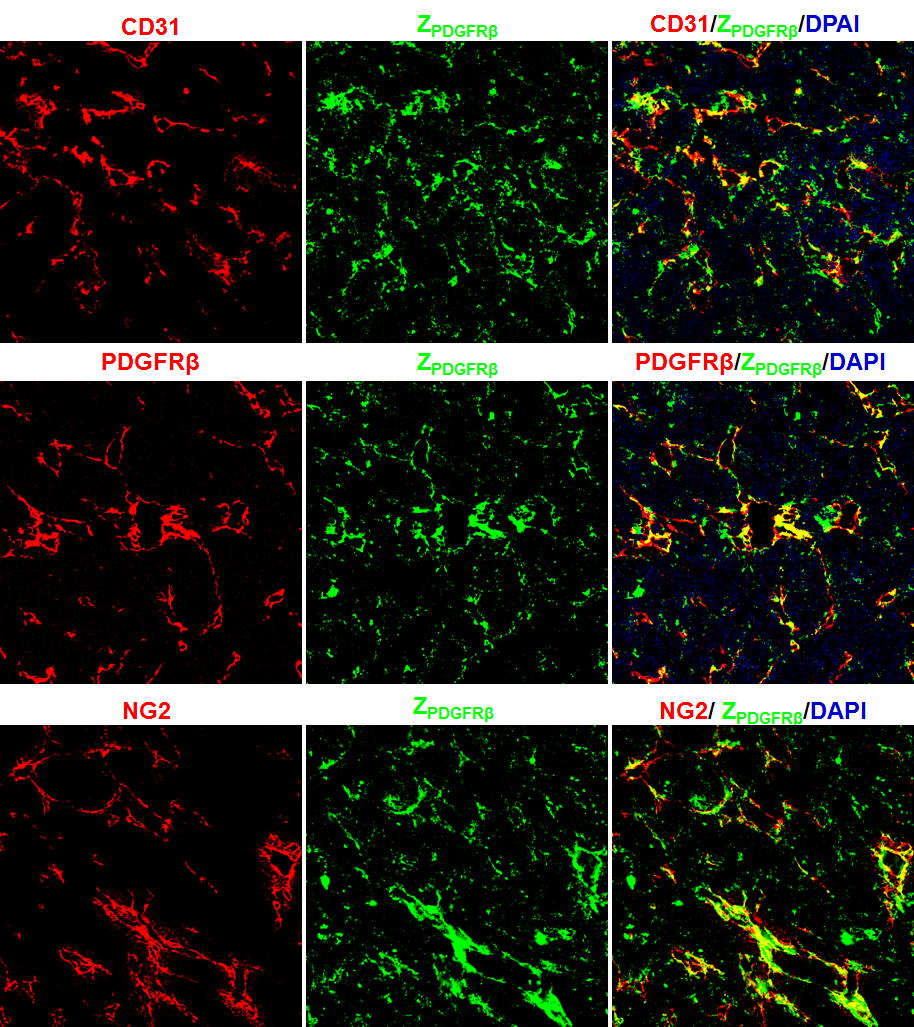


**Figure S1** Co-localization of the Z_PDGFRβ_ affibody with CD31, PDGFRβ, and NG2 in tumor tissues. FAM-labeled Z_PDGFRβ_ affibody was intravenously injected into mice bearing LS174T tumor grafts. Approximately 4 h later, tumor grafts were removed and sectioned under freezing conditions, followed by staining with antibody against CD31, PDGFRβ, or NG2. The nuclei were visualized by DAPI staining. Original magnification ×200.

**
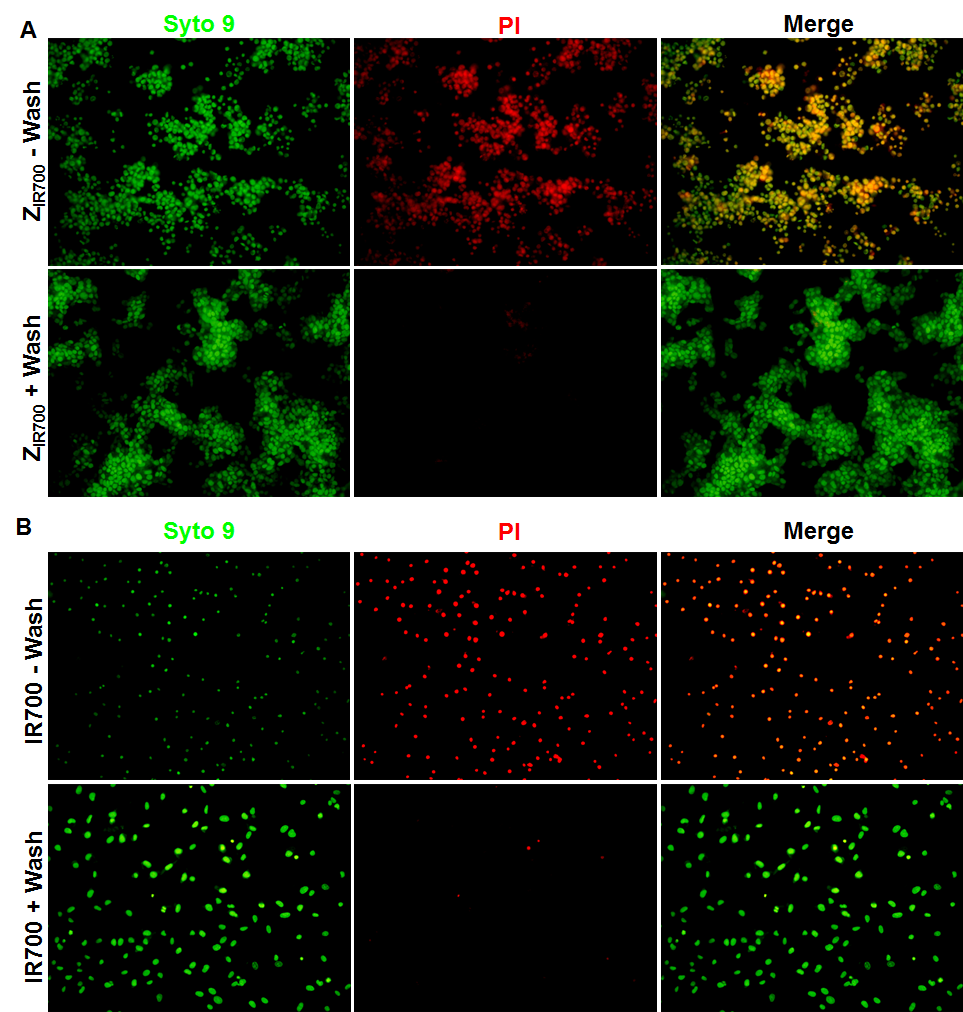
**

**Figure S2** Photocytotoxicity of IR700 and Z_IR700_ in cells. (A) Photocytotoxicity of IR700 in pericytes. Cells were preincubated with IR700 for 1 h prior to illumination and washed with PBS (IR700+wash) or not washed (IR700-wash). (B) Photocytotoxicity of Z_IR700_ in PDGFRβ-negative LS174T tumor cells washed with PBS (Z_IR700_+wash) or not washed (Z_IR700_-wash). Original magnification ×200.

**
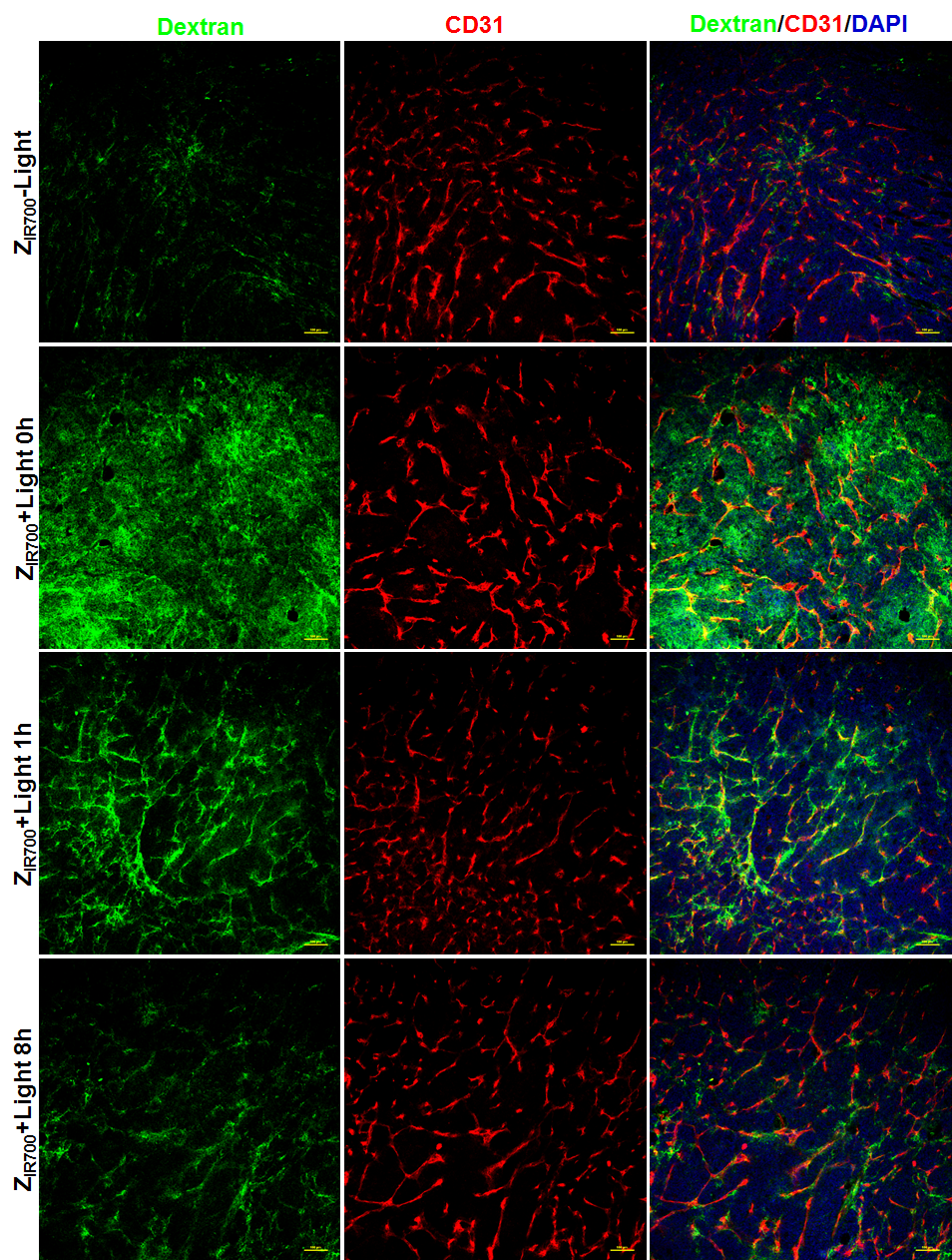
**

**Figure S3** Permeability of tumor blood vessels visualized by the leakage of dextran. After treatment with Z_IR700_-mediated PDT, mice bearing LS174T tumor grafts were intravenously injected with FITC-dextran at different times (0-8 h) post-illumination. Approximately 20 min post-injection of dextran, the tumor grafts were removed and sectioned under freezing conditions followed by visualization of the blood vessels with antibody against CD31. DAPI was used to visualize the nuclei. Original magnification ×100.

**
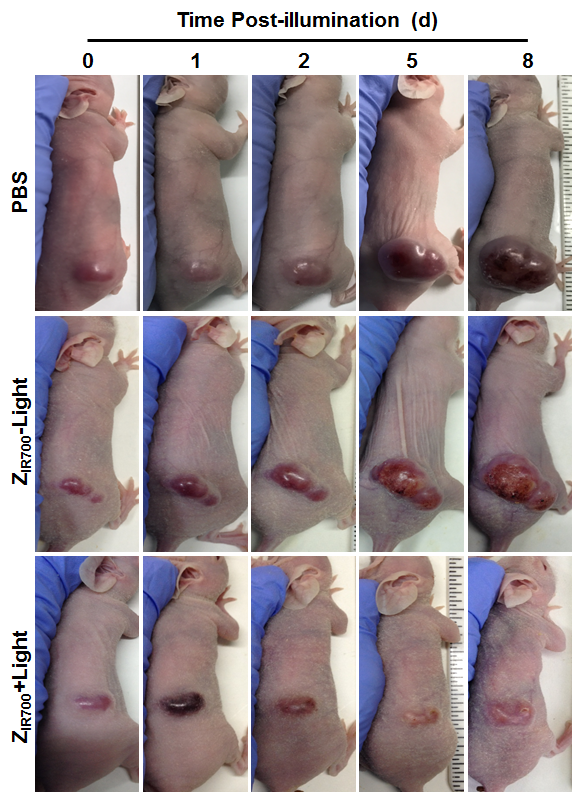
**

**Figure S4** Z_IR700_-mediated PDT in mice bearing LS174T tumor grafts. Mice bearing LS174T tumor grafts were intravenously injected with Z_IR700_ followed by illumination (Z_IR700_+Light) or not (Z_IR700_-Light). Mice in the control group were treated with PBS. Representative photographs of tumor grafts at different times (0-8 d) post-illumination are shown.
